# *Aspergillus* endocarditis diagnosed by fungemia plus serum antigen testing

**DOI:** 10.1016/j.mmcr.2018.10.004

**Published:** 2018-10-25

**Authors:** Timothy J. Hatlen, Scott G. Filler, Arnold Bayer, Sonia Shah, Shivani Shodhan, Tam T. Van

**Affiliations:** a Division of Infectious Diseases, Los Angeles Biomedical Research Institute, Harbor-UCLA Medical Center, Torrance, CA 90509, USA; b Division of Cardiology, Harbor-UCLA Medical Center, Torrance, CA 90509, USA; c Department of Pathology, Harbor-UCLA Medical Center, Torrance, CA 90509, USA; d Department of Pathology and Laboratory Medicine, David Geffen School of Medicine, University of California-Los Angeles, Los Angeles, CA 90095, USA

**Keywords:** Fungal Endocarditis, Aspergillus fumigatus, Fungemia

## Abstract

Fungal endocarditis remains an uncommon clinical diagnosis, though is likely to become more frequent due to the global increase in transplantations and cardiac valvular surgery. A case of prosthetic valve endocarditis due to Aspergillus fumigatus is described that was diagnosed with serologic fungal markers and confirmed with positive blood cultures, an uncommon finding.

## 1 Introduction

Fungal endocarditis (FE) is a rare, but emerging clinical entity encompassing 2–5% of all cases of infective endocarditis. *Candida* species account for most of cases (53–68%), followed by *Aspergillus* species (20–25% of cases) [1]. *Candida* endocarditis is frequently diagnosed by a combination of echocardiography and positive blood cultures. In contrast, the diagnosis of *Aspergillus* endocarditis is challenging because blood cultures are frequently negative, with most cases diagnosed by either histopathologic examination of the resected valve or postmortem [2]. More recently, the use of serodiagnostic markers is a promising strategy to more rapidly diagnose *Aspergillus* endocarditis [3]. Herein, we report an unusual case of *Aspergillus fumigatus* prosthetic valve endocarditis presumptively diagnosed by serum (1, 3)-β-D-glucan (BDG) and galactomannan (GM) testing, and subsequently confirmed by positive blood cultures, a rare scenario.

## 2 Case

A 58-year-old male presented (day −120) with symptoms of congestive heart failure that developed over a 2-week period. Transthoracic echocardiography was notable for a 30% ejection fraction, moderate to severe aortic regurgitation and severe mitral regurgitation. Because his symptoms were refractory to medical therapy, he underwent transesophageal echocardiography for anticipated valvular surgery, which revealed 4–6 mm mobile echodensities on the mitral valve. The patient denied any constitutional symptoms, fevers or chills, weight loss, or skin lesions. He was born in Mexico and had exposure to livestock as a child. He had lived in the USA for the past 28 years and worked as a gardener in the greater Los Angeles area with no travel outside the city for more than 20 years. He denied any exposure to pets, history of homelessness, or sick contacts. His blood cultures were negative, as was serologic evaluation for *Brucella*, *Bartonella*, and *Coxiella*. The patient was prescribed a 6-week course of ceftriaxone and doxycycline. During this time, he underwent aortic and mitral valve surgery with bioprosthetic placement. Bacterial sequencing using broad range PCR primers (completed by the University of Washington department of laboratory medicine) of his native valve was negative for bacterial pathogens. Pathology of the valve reported papillary fibroelastoma.

The patient returned one month following valve surgery (day −70) with fevers, chills and reported night sweats for 3 weeks. He had completed his prior antibiotics. Empiric therapy for presumed bacterial prosthetic valve endocarditis was initiated. Transesophageal echocardiography demonstrated normal hemodynamics of his prostheses, a small 0.4 cm mobile echodensity was noted on the mitral valve. His EKG showed a new third-degree AV block. However, 3 blood culture sets prior to antibiotic administration were negative, and the patient was asymptomatic without documented fevers in the hospital. Since he had only 1 major criterion (positive echocardiogram for possible “vegetations”) plus one minor criterion (underlying valvular disease) by the Duke Modified Criteria [4], it was felt that he likely had noninfectious vegetative endocarditis perhaps due to post-valvular surgery thrombus. Empiric antibiotic therapy was stopped and the patient remained clinically stable and afebrile over subsequent days of monitoring. A pacemaker was placed.

One month later (day 0), the patient presented with 3 weeks of fevers, chills, night sweats, and dyspnea on exertion. He reported no cough, new skin lesions, and the sternal wound was asymptomatic. He was afebrile and his examination was notable for a generalized frail appearance, a new diastolic murmur appreciated best at the heart apex radiating to the axilla and bibasilar rales. He had leukocytosis of 15,500/mm^3^ and elevation of his creatinine to 0.88 mg/dL (baseline 0.55 mg/dL). Blood cultures were drawn, and patient was started on empiric vancomycin, rifampin, and gentamicin for presumed prosthetic valve bacterial endocarditis.

**Fig. 1:**
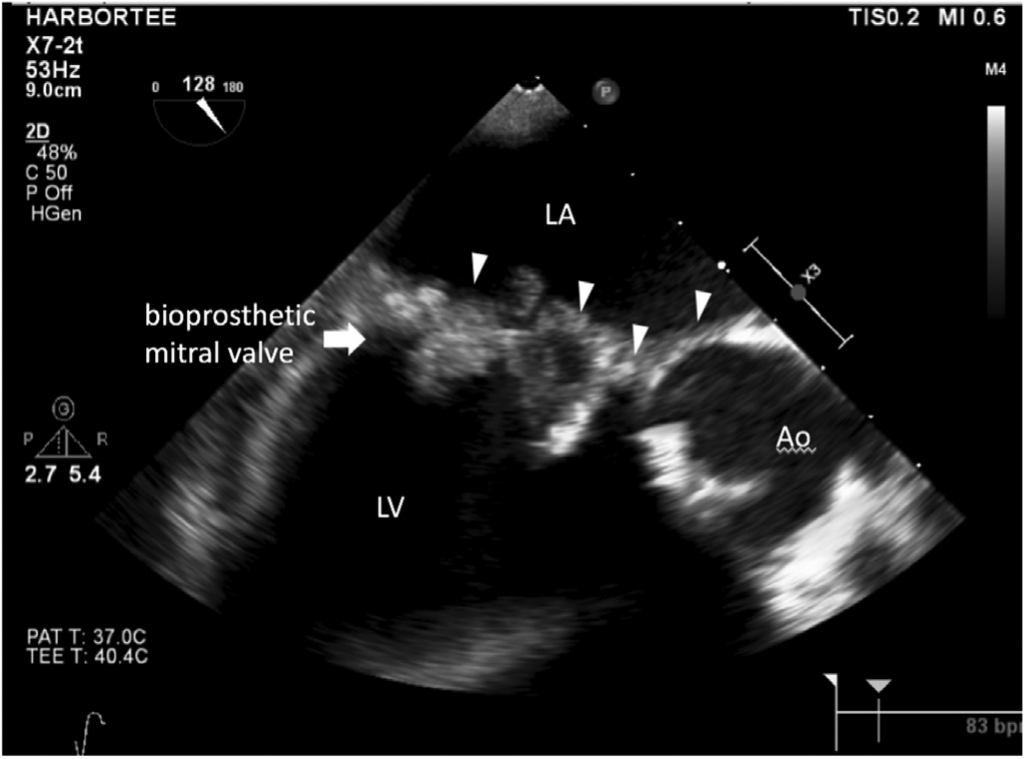
Transesophageal echocardiogram, mid-esophageal view. Shows 33 mm bioprosthetic mitral valve with extensive large, bulky vegetations encasing the leaflets. There is valve dehiscence and extension to the aortomitral curtain and aortic root (arrows). Mild.

**Fig. 2:**
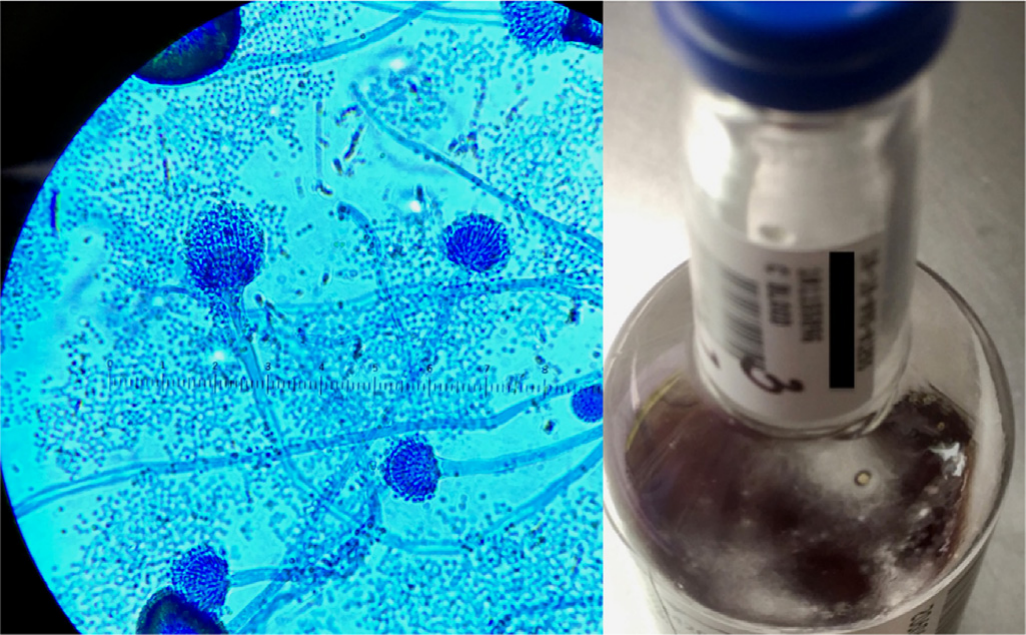
Left: *Aspergillus fumigatus* from blood. 600x magnification. Lactophenol blue dye. Right: Visible fungal ball within blood culture bottle.

A transesophageal echocardiogram demonstrated new bulky vegetations of up to 3.3 cm on the mitral valve encasing the leaflets, with a 0.8 cm highly mobile component, with sparing of the aortic valve and pacemaker leads. The infection extended to the aorto-mitral curtain and aortic root (Fig. 1). Wedge-shaped densities, probably infarcts, of the spleen and kidneys were visible by abdominal CT scan; in addition, a 2.9 cm occipito-parietal intraparenchymal hemorrhage was seen on head CT scan. By day 3 of hospitalization, the admission blood cultures remained sterile, although the patient started spiking fevers. After a multi-disciplinary conference, he was started empirically on liposomal amphotericin B 5 mg/kg daily. The following day, the serum BDG was reported as > 500 pg/ml (negative cutoff < 59 pg/ml), and the serum GM EIA index value was elevated at 2.24 (negative cutoff index < 0.5). As a result, the patient was administered voriconazole 4 mg/kg IV twice daily in addition to liposomal amphotericin B 10 mg/kg/d. He was also continued on vancomycin and cefepime. The rifampin was discontinued due to the concern that it would lower the serum voriconazole levels. Valvular surgery was not performed due to worsening of the intraparenchymal hemorrhage. On day 11, fungal balls were observed in the patient's admission blood culture bottles (Fig. 2). The mold that subsequently grew on solid media showed phenotype consistent with *Aspergillus* and identification was later confirmed as *Aspergillus fumigatus* (Fig. 2). With development of transaminitis and acute kidney injury, the liposomal amphotericin B, vancomycin and cefepime were discontinued, and micafungin 100 mg IV daily was added to the voriconazole. Unfortunately, although the patient's organ failure improved, surgical intervention was determined to be too high-risk. On day 17, the patient died from a presumed massive stroke. Serum obtained on day 16 showed a BDG > 500 pg/ml and GM of 2.63. An autopsy was not obtained.

## 3 Discussion

Fungal endocarditis (FE) remains an uncommon clinical diagnosis, though is likely to become more common due to the global increase in transplantations and cardiac valvular surgery. Although only 2–5% of all cases of endocarditis are caused by fungi, this form of endocarditis, especially when caused by *Aspergillus,* is associated with very high morbidity and mortality rates as compared to bacterial endocarditis [5], [6], [7]. The high mortality associated with fungal endocarditis is related to: i) its frequent occurrence in debilitated or immunocompromised hosts; ii) frequently negative blood cultures; iii) delayed diagnoses; and iv) the frequent failure of antifungal therapy alone, in the absence of valve replacement. In a review of 270 cases of fungal endocarditis, only 18% of providers had a “fungal etiology” in the initial differential diagnosis. Also, the average time until diagnosis was 17 days, and the mean survival of patients after initiating antifungal therapy was only 11 days for *Aspergillus* endocarditis vs. 690 days for *Candida* endocarditis [8]. These data underscore the imperative need for accurate non-invasive diagnostic tests to prompt the early initiation of antifungal therapy, prior to the development of valve dysfunction, heart failure, and/or embolic phenomena. Although the case report presented herein resulted in a suboptimal clinical outcome, it emphasizes the use of serum antigen testing to trigger the initiation of empiric antifungal therapy in cases of “culture-negative” endocarditis. This report also highlights that positive blood cultures, although rare in *Aspergillus* endocarditis, can be an important diagnostic finding.

Large literature reviews of FE case reports have been performed, covering the years 1965–1995 [8], 1995–2000 [1] and 2003–2011 [2]. Also, there have been several large reviews focusing specifically on *Aspergillus* endocarditis from 1950 to 2010 [6], 1964–2005 [7], and 2003–2009 [3]. Autopsy studies demonstrate an increase in incidence of FE from 2.5% in 1970–1985–11% in 1986–2008 [9]. Pierrotti et al. [1], reported 40 cases of FE due to molds, of which 28 (71%) were *Aspergillus* spp. Among the cases of *Aspergillus* endocarditis, 54% were due to *A. fumigatus* (as in our case), 18% *Aspergillus terreus*, 7% *Aspergillus niger*, and *7% Aspergillus flavus*.

In a review of 267 cases of FE, 97% of the patients had at least one known key risk factor, of which prior valve surgery (54%) was most common [8]. In a compilation of 34 patients with *Aspergillus* endocarditis, 74% had history of prior cardiac valve surgery and 40% had undergone organ transplantation [3]. A case series of patients with native valve *Aspergillus* endocarditis found that approximately two-thirds of the patients were immunocompromised due to steroids, malignancy, and/or solid organ transplantation; only 3% had HIV/AIDS [10]. By contrast, in a study of 164 patient who developed *Aspergillus* endocarditis following cardiac surgery, none were immunocompromised and the infection was likely acquired peri-operatively [7]. The author's surmise the case presented is most aligned with peri-operative acquisition of the initial prosthetic valve placement.

The diagnosis of *Aspergillus* endocarditis is difficult. Clinical symptoms are non-specific, although fever occurs in more than half of patients. The most common signs include large vegetations on echocardiography and a high rate of peripheral embolization (53%) at presentation [6]. The antemortem diagnosis of *Aspergillus* endocarditis is usually made by culture and histopathologic analysis of resected valvular tissue or peripheral embolic sites [3], [6]. In approximately 45% of cases, the diagnosis is made postmortem [2]. Although automated blood culture bottles have been shown to be capable of supporting growth of *Aspergillus* spp. [11], blood cultures are usually negative even in patients with intravascular infection and evidence of peripheral embolic disease. For example, of 114 patients with *Aspergillus* endocarditis, the organism was isolated from blood culture in only 7 (6.7%) cases [7]. It has been hypothesized that when viable *Aspergillus* hyphae enter the bloodstream, they are endocytosed by endothelial cells, leading to subsequent endothelial cell injury and vascular thrombosis, which sequesters the organism. The only fungal elements that circulate in the bloodstream are non-viable [12].

Nevertheless, as in our case, the growth of *Aspergillus* spp. in a blood culture from a patient with suspected FE provides invaluable information: i) it facilitates early diagnosis prior to valve surgery; ii) allows for identification of the organism at the species level (which cannot be done by histopathology [13]); and iii) provides an isolate for antifungal susceptibility testing. The latter benefit is particularly important because of the well-documented rise in azole resistance among *Aspergillus* spp. and the rising incidence of infection caused by less common *Aspergillus* spp. with intrinsic antifungal resistance [14].

Given the poor sensitivity of blood cultures, the use of non-culture-based serodiagnostic tests is a promising approach for the early recognition of *Aspergillus* endocarditis. In review of 20 cases of *Aspergillus* endocarditis, in which serum *Aspergillus* PCR, BDG and/or GM testing was performed, PCR was positive in 7 of 7 patients tested, serum BDG was positive in 6 of 7 (85.7%) patients and GM in 10 of 16 (62.5%). In 4 of 20 patients (20%), all three tests were negative [15]. The underlying risk factors for these patients were prior cardiac surgery (n = 3), transplantation (n = 2) and malignancy (n = 2). Reporting bias contributes to a substantial limitation in interpretation of these results. BDG and GM are recommended in the workup for FE. Neither BDG or GM are specific to *Aspergillus* and although *Aspergillus* PCR of the blood has yielded promising results in patients with *Aspergillus* endocarditis, recommendation for routine use in clinical practice remains debatable due to question on its use in clinical diagnosis and the lack of standardization on testing methodology [16].

The recommended treatment for empiric coverage of FE is liposomal amphotericin B 3–5 mg/kg/day [5]. For treatment of *Aspergillus* endocarditis, voriconazole 4 mg/kg twice daily is recommended [16]. The benefit of adding amphotericin B or an echinocandin to voriconazole is uncertain, although in a subset of patients with invasive aspergillosis and an elevated serum GM, those receiving combination therapy with voriconazole and anidulafungin had a lower 6-week mortality relative to patients who received voriconazole alone [17]. We opted to treat our patient with combination therapy because of concern that we would be unable to achieve therapeutic voriconazole levels due to the recent administration of rifampin for presumed bacterial prosthetic valve endocarditis [18]. Indeed, after 1 week of therapy, the patient's serum voriconazole level was subtherapeutic at 1.2 µg/ml. Currently, it is recommended that all patients with FE receive valve replacement surgery whenever feasible. However, this recommendation is not based on results from prospective studies. For patients who survive FE, lifelong oral antifungal suppressive therapy is recommended because of the high rate of relapse, even after apparent cure [16].

Although the patient ultimately died, this case demonstrates the utility of obtaining serum BDG and GM in a patient with prosthetic valve endocarditis in whom bacterial blood cultures are negative. While *A. fumigatus* eventually grew in blood cultures from this patient, relying on blood culture alone to make the diagnosis would have delayed therapy for 7 days. Furthermore, in approximately 93% of patients with *Aspergillus* endocarditis, blood cultures are never positive [7]. In the future, *Aspergillus* blood PCR may provide an even more rapid way to diagnose this frequently deadly disease.
